# Massive haematemesis as the first manifestation of superior mesenteric and portal vein thrombosis in a healthy young adult

**DOI:** 10.1093/jscr/rjaf985

**Published:** 2025-12-13

**Authors:** Saleh Yasin, Abdelrahman Mohammed, Ahmad Zohud, Amro Odeh, Ayman Altarifi

**Affiliations:** Surgery Department, Al Istishari Hospital, Al-Rehan Street, Al-Rehan Neighborhood, Ramallah P623, Palestine; Internal Medicine Department, Al Istishari Hospital, Al-Rehan Street, Al-Rehan Neighborhood, Ramallah P623, Palestine; Department of Medicine, Faculty of Medicine and Health Sciences, An Najah National University, Rafidia, Nablus P400, Palestine; Department of Medicine, Faculty of Medicine and Health Sciences, Al-Quds University, University street, Abu Dis, Jerusalem P144, Palestine; General Surgery, Endoscopy and Morbid Obesity Surgery, Al Istishari Hospital, Al-Rehan Street, Al-Rehan Neighborhood, Ramallah P623, Palestine

**Keywords:** superior mesenteric vein thrombosis, portal vein thrombosis, haematemesis, intestinal ischaemia, small-bowel resection, protein S deficiency

## Abstract

Superior mesenteric vein (SMV) and portal vein thrombosis are rare but serious causes of intestinal ischaemia, and haematemesis as an initial presentation is highly uncommon. We report the case of a 39-year-old previously healthy man who presented with acute haematemesis following diffuse abdominal pain. Contrast-enhanced computed tomography revealed complete thrombosis of the portal and SMV with ischaemic small bowel. Emergency laparotomy confirmed extensive jejunal and ileal gangrene, requiring near-total small-bowel resection with end jejunostomy. Postoperatively, the patient required intensive care with anticoagulation, parenteral nutrition, and broad-spectrum antimicrobials. His course was complicated by high jejunostomy output, catheter-related bloodstream infection, and bilateral pulmonary embolism despite anticoagulation. Thrombophilia testing revealed low Protein S levels. With continued supportive care, he gradually improved, his inflammatory markers normalized, and he was discharged haemodynamically stable with plans for long-term nutritional and anticoagulation support. This case illustrates a rare presentation of SMV and portal vein thrombosis, underscoring the importance of early diagnosis and intervention.

## Introduction

Portal vein (PV) thrombosis (PVT) involves the formation of a thrombus in the PV and its branches, potentially extending to the splenic and superior mesenteric vein (SMV) [1]. Genetic mutations in antithrombin, protein C, and protein S are frequently associated with PVT and SMVT. These mutations can lead to idiopathic thrombosis, where no other underlying cause is identified [2]. Conditions such as cirrhosis, hepatobiliary malignancies, and inflammatory or infectious diseases can predispose individuals to PVT [3]. In cirrhosis, decreased blood flow due to portal hypertension increases the risk of thrombosis [2].

Contrast-enhanced computed tomography (CT) scans are crucial for diagnosing PVT and SMVT. They help visualize the extent of thrombosis and assess for complications such as intestinal ischaemia. Patients often present with abdominal pain, which can be acute and severe. In some cases, symptoms may be non-specific, making early diagnosis challenging. Haematemesis is an uncommon presentation of SMVT and PVT [4]. Haematemesis may occur if there is significant portal hypertension leading to esophageal varices [5].

Treatment of SMVT and PVT often involves anticoagulation to prevent further thrombus formation and to promote recanalization of the veins [6]. In some cases, direct thrombolytic therapy or surgical intervention may be necessary, especially if there is significant bowel ischaemia or variceal bleeding [1]. We report the case of a 39-year-old man who presented with massive upper gastrointestinal bleeding and was subsequently found to have acute portal and SMV thrombosis with ischaemic small bowel.

## Case presentation

A 39-year-old previously healthy man presented with acute upper gastrointestinal bleeding. Three days before admission, he developed progressive epigastric pain associated with nausea and a single episode of non-bilious vomiting. He initially received symptomatic treatment at a local clinic, but his pain worsened and became diffuse. The following day, he had haematemesis of fresh blood and was found to have a rigid, distended abdomen. Contrast-enhanced CT suggested mesenteric venous ischaemia, and he was managed conservatively with fluids, antiemetics, and enoxaparin. On referral to our center, he experienced massive haematemesis and was haemodynamically unstable with tachycardia and hypotension, necessitating urgent transfer.

**Figure 1:**
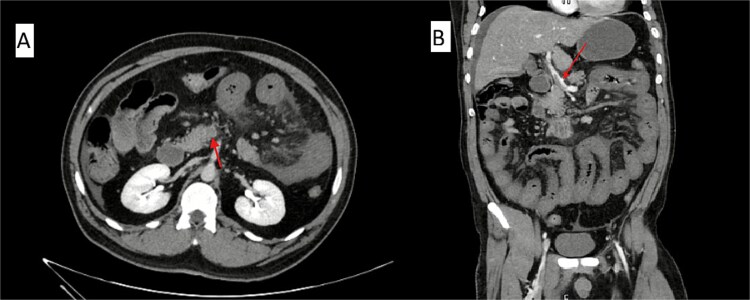
Contrast-enhanced abdominal CT scan showing acute mesenteric venous thrombosis. (A) Axial view demonstrating complete thrombosis of the superior mesenteric vein (arrow). (B) Coronal view confirming thrombus extension into the portal venous system (arrow).

On arrival, his haemoglobin was 7.2 g/dl, WBC 17.5 × 10^9^/L, and CRP 321 mg/L. Electrolytes revealed mild hyponatremia, hypocalcemia, and hypophosphatemia, with preserved renal function. Coagulation studies showed mildly prolonged prothrombin time. Upper endoscopy demonstrated active bleeding beyond the duodenum without an identifiable source. Repeat CT confirmed complete thrombosis of the portal and superior mesenteric veins with small-bowel ischaemia (Fig. 1). Emergency laparotomy revealed extensive jejunal and ileal gangrene, requiring near-total small-bowel resection with end jejunostomy (Video 1).

Postoperatively, he was admitted to the ICU, where he received therapeutic enoxaparin, broad-spectrum antibiotics (meropenem and fluconazole), and structured fluid and electrolyte replacement. His jejunostomy output ranged from 1.5 to 2.2 L/day, necessitating parenteral nutrition and high-dose loperamide. Serial labs demonstrated persistent inflammation (C-reactive protein (CRP) 321 → 171 → 121 mg/L) with stable renal function and mild hypoalbuminemia. Drain outputs were monitored, and dressings were changed daily.

On postoperative Day 10, the patient developed acute chest pain and hypoxemia. CT pulmonary angiography confirmed bilateral pulmonary embolism despite therapeutic anticoagulation. Haematology was consulted, and extended thrombophilia testing was initiated, revealing low Protein S, with additional tests pending. He remained on full-dose anticoagulation.

His ICU course was further complicated by a catheter-related bloodstream infection with gram-positive cocci, managed successfully with line removal and vancomycin. Over the following days, his inflammatory markers improved (CRP decreased to 36 mg/L). He was extubated, transitioned to clear liquids, encouraged to ambulate, and prepared for a step-down transfer once he was stable. At discharge, he was haemodynamically stable, with ongoing nutritional support needs and plans for long-term central venous access.

## Discussion

Mesenteric vein thrombosis (MVT) is a rare but serious cause of intestinal ischaemia, accounting for ~5%–15% of all mesenteric ischaemia cases [3]. The usual first symptom is sudden abdominal pain, while haematemesis is usually seen in portal hypertension and variceal bleeding, not in MVT [4, 5]. Haematemesis as the first symptom makes this case unusual; also, the patient was young and previously healthy.

Most patients with MVT have risk factors like cancer, cirrhosis, or inherited clotting problems [2, 3]. Still, about one in four cases happen without any apparent cause [2]. Our patient also had no known risk factors, indicating that MVT can occur in individuals who are otherwise healthy.

Diagnosing MVT can be hard because early symptoms can look like more common stomach problems. Abdominal pain with haematemesis might first suggest peptic ulcer disease, variceal bleeding, or pancreatitis. This can easily delay the correct diagnosis, which is dangerous because bowel infarction can develop quickly. Contrast-enhanced CT scan is the best test, with high diagnostic accuracy [3, 4]. In our case, CT confirmed the diagnosis and allowed urgent surgery, showing why CT should be done early when intestinal ischaemia is suspected.

The primary treatment is anticoagulation, which is associated with better outcomes and venous recanalization in many patients [7]. Other options include endovascular therapy, such as catheter-directed thrombolysis or thrombectomy, in selected cases [6, 8], and surgery if bowel infarction has already developed. Our patient needed a large part of his small bowel removed and later had a pulmonary embolism even while on anticoagulation, which has been described in other series [6, 8]. This indicates that new or recurrent blood clots can occur, and it is essential to arrange long-term follow-up and testing for underlying clotting disorders.

In conclusion, this case shows that MVT can sometimes present in unusual ways, such as haematemesis, even in young patients with no clear risk factors. Doctors should consider MVT in patients with abdominal pain and gastrointestinal bleeding. Early CT imaging and testing for thrombophilia are crucial, and timely anticoagulation, along with careful follow-up, are essential to reduce the risk of complications.

## Supplementary Material

Supplementary_Video_1_rjaf985

WhatsApp_Video_2025_09_12_at_20_59_40_rjaf985

## Data Availability

All data generated or analyzed during this study are included in this article. Further inquiries can be directed to the corresponding author.
