# Supplementary material for: Massive haematemesis as the first manifestation of superior mesenteric and portal vein thrombosis in a healthy young adult
Source: J Surg Case Rep. 2025 Dec 13;2025(12):rjaf985. doi: 10.1093/jscr/rjaf985 (PMC12701568; doi:10.1093/jscr/rjaf985)
Supplement: Supplementary_Video_1_rjaf985 [file supplementary_video_1_rjaf985.docx]

**Video 1. Intraoperative findings of acute mesenteric ischemia.** The video demonstrates the surgical exploration revealing extensive jejunal and ileal gangrene secondary to superior mesenteric and portal vein thrombosis. Resection of the necrotic small bowel was performed, followed by the creation of an end jejunostomy.
